# Geriatric Nutritional Risk Index Predicts Adverse Outcomes in Human Malignancy: A Meta-Analysis

**DOI:** 10.1155/2019/4796598

**Published:** 2019-11-19

**Authors:** Guo-yue Lv, Lin An, Da-wei Sun

**Affiliations:** ^1^Department of Hepatobiliary and Pancreatic Surgery, The First Hospital of Jilin University, Jilin University, Changchun, 130021 Jilin, China; ^2^Department of Hand Surgery, China-Japan Union Hospital of Jilin University, Jilin University, Changchun, 130033 Jilin, China

## Abstract

**Background:**

Geriatric Nutritional Risk Index (GNRI) has been widely used to assess the nutritional status in a variety of human pathological conditions, but the prognostic value of the GNRI in malignancies has not been evinced.

**Methods:**

Relevant studies updated on Jul 27, 2019, were retrieved in available databases, including PubMed, Web of Science, Cochrane library, Chinese CNKI, and Chinese Wan-fang. Hazard ratios (HRs) and 95% confidence intervals (CIs) were extracted and pooled by using STATA 14.

**Results:**

A total of 15 studies involving 8,046 subjects were included in this meta-analysis. Meta-analysis results evinced that low GNRI was associated with poor OS (HR = 1.95, 95% CI: 1.49-2.56, *p* ≤ 0.001), poor CSS (HR = 1.81, 95% CI: 1.49-2.19, *p* ≤ 0.001), poor DFS (HR = 1.67, 95% CI: 1.28-2.17, *p* ≤ 0.001), and poor PFS (HR = 1.68, 95% CI: 1.28-2.21, *p* ≤ 0.001), and the correlation of GNRI with OS was not changed when stratified by possible confounding factors, suggesting that malignancy patients with low GNRI would suffer from reduced survival rate and increased recurrence rate. Moreover, low GNRI was also associated with postoperative complications in malignancies.

**Conclusions:**

In summary, GNRI is associated poor prognosis in human malignancies, and GNRI should be used as a predictive indicator of adverse outcomes during malignancy treatment.

## 1. Introduction

In 2005, Bouillanne et al. created a new index of malnutrition, called the Geriatric Nutritional Risk Index (GNRI), which is based on three parameters: height, body weight, and serum albumin level. The GNRI was calculated as follows: [1.489 × albumin (g/L)] + [41.7 × (body weight/ideal body weight)]. The ratio of body weight to ideal body weight was set to 1 when the patient's body weight exceeded the ideal body weight [1]. This study firstly reported that GNRI was a simple and accurate tool for predicting the risk of morbidity and mortality in hospitalized elderly patients [1]. Thereafter, the GNRI has also been widely used to assess the nutritional status and also reported to be associated with adverse outcomes in a variety of human pathological conditions. For instance, the low-GNRI group was reported to be associated with a higher rate of postoperative complications and longer length of hospital stay when compared with those in the high-GNRI group in patients who underwent abdominal surgery [2], an increased risk of all-cause and cardiovascular mortality in chronic hemodialysis patients [3], all-caused death and cardiac death in patients who underwent percutaneous coronary intervention [4], poor prognosis (including higher risk of mortality, metastatic infection, and organ dysfunction) in pyogenic liver abscess patients [5], short-term hospital mortality in older patients with sepsis [6], and long-term survival plus cardiovascular/limb events in patients with peripheral arterial disease [7].

Malnutrition is a common problem among cancer patients, and cancer-associated malnutrition is associated with increased morbidity and mortality [8]. Meanwhile, the occurrence and development of cancers have been shown to be associated with aging [9] and the elderly subjects are considered to be susceptible with more risk of nutritional problems [10]. As a simple and well-established nutritional assessment tool, the GNRI is also reported to be a significant prognostic factor for various malignancies recently.

However, the prognostic significance of GNRI on human malignancies has not been thoroughly clarified. Therefore, this meta-analysis was conducted to verify the prognostic role of GNRI in human malignancies based on available evidence.

## 2. Materials and Methods

### 2.1. Search Strategy

Our literature search strategy was performed according to the preferred reporting items for systemic reviews and meta-analysis (PRISMA) statement criteria [11]. We retrieved literatures about the prognostic significance of the GNRI in patients with malignancies, which were published before July 27, 2019, in available databases, including *PubMed*, *Web of Science*, *Cochrane library*, *Chinese CNKI*, and *Chinese Wan-fang*. We used the following search words: “*Geriatric Nutritional Risk Index*,” “*GNRI*,” and “*cancer*,” “*carcinoma*,” “*leukemia*,” and “*lymphoma*.” The search strategy in Cochrane was “*Geriatric Nutritional Risk Index in Abstract OR GNRI in Abstract*.” The search strategy in PubMed was “(*Geriatric Nutritional Risk Index[Title/Abstract]) OR GNRI[Title/Abstract]*.” The search strategy in Web of Science was “*TS=(Geriatric Nutritional Risk Index in Abstract OR GNRI)*.” The reference lists of retrieved literatures were also screened to identify more potential studies.

### 2.2. Inclusion and Exclusion Criteria

Studies included in this meta-analysis were required to meet all of the following criteria: (1) reporting the prognostic role of the GNRI in human malignancy; (2) analyzing prognostic outcomes, including overall survival (OS), cancer-specific survival (CSS), disease-free survival (DFS), or progression-free survival (PFS); and (3) providing the hazard ratio (HR) and 95% its confidence interval (CI) for prognosis (or calculable according to data provided in the original literature). The studies were excluded if they met any of the following items: (1) case report, (2) review article, (3) redundant publication, and (4) HR and 95% CI unacquirable.

### 2.3. Data Extraction

This procedure was conducted by two authors (Da-wei Sun and Lin An), and disagreements were resolved by consensus among all the participating authors. The hazard ratios (HRs) and 95% confidence intervals (CIs) for prognosis were our main concern. When the prognostic results were provided in the Kaplan-Meier curve, Engauge Digitizer 4.1 was used to read and calculate the number of death/recurrence in each group. Then according to the total numbers of observed deaths/recurrences and the number of samples in each group, we calculated the HR and 95% CI by following Tierney et al.'s method [12]. The other important items included the 1^st^ author information, publication year, patient country, cancer species, sample capacity, dividing line for GNRI, HR origin, analytic methodology, and follow-up interval. For studies based on the same study center, we collected data from the study with the largest sample size. Here, we declared that the data extraction method in this part was nearly the same as that used in our team's previously published meta-analysis researches.

### 2.4. Statistics Analysis

Hazard ratios (HRs) and 95% confidence intervals (CIs) were used to compare the prognostic outcomes, including OS, CSS, DFS, and PFS. Chi-square test and *I*^2^ were used to check the interstudy heterogeneity, with significance set at *p* < 0.1 or *I*^2^ > 50%. A random effects model was employed in case of significant heterogeneity; otherwise, a fixed effects model was applied. Publication bias was examined by both Begg's and Egger's tests [13, 14]. In this meta-analysis, a *p* value < 0.05 was considered significant. Meanwhile, we performed a sensitivity analysis by removing each study to evaluate the effect of an individual study on the overall pooled HRs. Here, we also declared that the statistics analysis method in this part was nearly the same as that used in our team's previously published meta-analysis researches.

## 3. Results

### 3.1. Systemic Review for Included Studies

**Figure 1:**
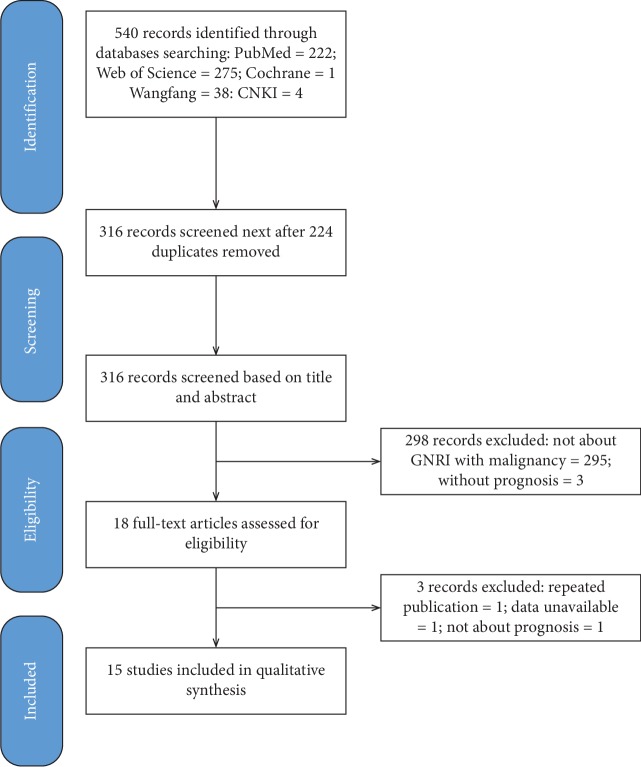
Literature selection process by following PRISMA guidelines in our meta-analysis.

Figure 1 illustrates the searching procedure of potential included studies. In the end, a total of 15 studies with 8,046 cases were identified through systemic search [15–29]. Five studies were based on patients with hematological malignant tumors [15–19], 4 studies were based on esophageal cancers [20–23], 2 studies were based on renal cell carcinoma [24, 25], and 4 studies were based on hepatocellular carcinoma, prostate cancer, pancreatic cancer, and lung cancer respectively [26–29]. Among these studies, two studies reported HRs for prognosis by two subsets in each study [23, 27]. On the basis of population origin, all the studies were based on Asian countries (1 in Korea, 9 in Japan, and 5 in China). The publication year happened between 2017 and 2019, the study sample size varied from 63 to 4,591, and the demarcation for GNRI ranged from 92 to 99.2. The other characteristics of the included studies, such as age, gender ratio, primary therapy, endpoint, source of HR, analytic method of HR, and follow-up interval, could be seen in Table 1.

### 3.2. Meta-Analysis for OS

**Figure 2:**
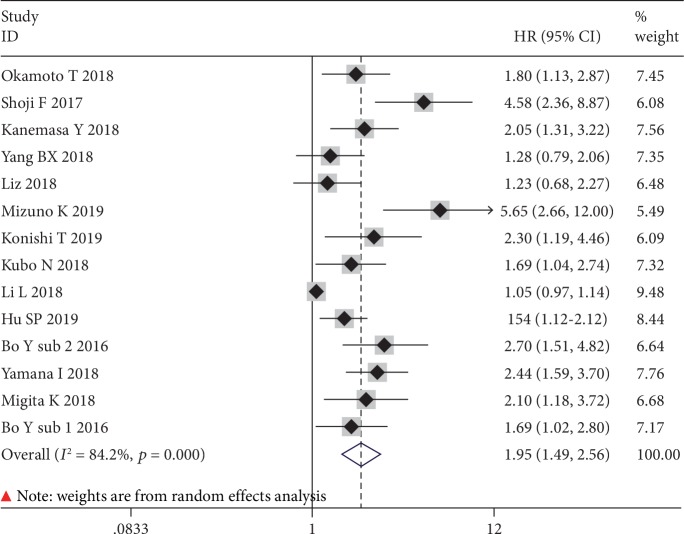
The forest plot for the effect of GNRI on OS in human malignancies.

The prognostic value of GNRI for OS was available in 13 studies (including one study consisting of two subcohorts) with 3,023 cases. On the basis of random effects model (*I*^2^ = 84.2%, *p* ≤ 0.001), the GNRI was significantly associated with OS (HR = 1.95, 95% CI: 1.49-2.56, *p* ≤ 0.001) (Figure 2). As a result, patients with low GNRI suffered from poor OS when compared with patients with normal GNRI.

### 3.3. Meta-Analysis for CSS

**Figure 3:**
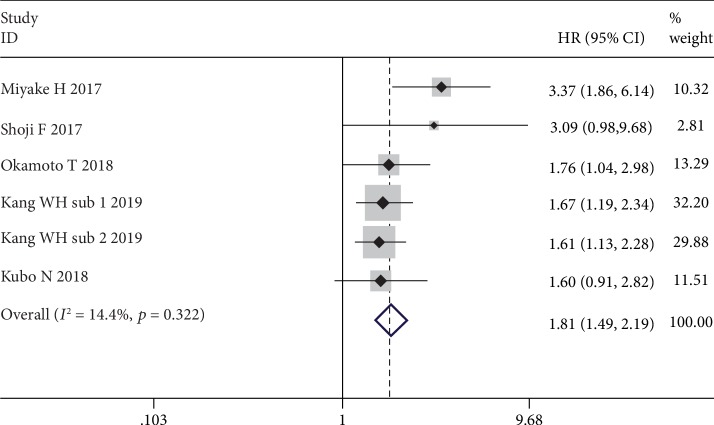
The forest plot for the effect of GNRI on CSS in human malignancies.

Five studies (including one study consisting of two subcohorts) with 5,743 cases reported the prognostic role of GNRI for CSS. According to a fixed effects model (*I*^2^ = 14.4%, *p* = 0.322), the GNRI was also significantly relevant to CSS (HR = 1.81, 95% CI: 1.49-2.19, *p* ≤ 0.001) (Figure 3). That is, patients with low GNRI suffered from poor CSS when compared with patients with normal GNRI.

### 3.4. Meta-Analysis for DFS

**Figure 4:**
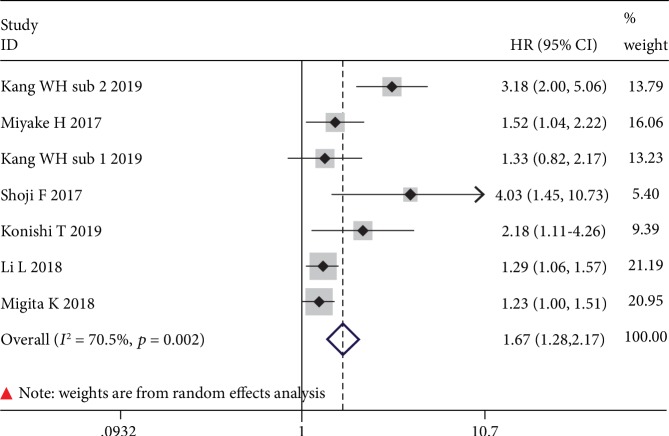
The forest plot for the effect of GNRI on DFS in human malignancies.

The correlation of the GNRI with DFS was available in 6 studies (including one study consisting of two subcohorts) with 5,625 cases. Meta-analysis results from a random effects model (*I*^2^ = 70.5%, *p* = 0.002) evinced that GNRI was also significantly correlated with DFS (HR = 1.67, 95% CI: 1.28-2.17, *p* ≤ 0.001) (Figure 4). That is, patients with low GNRI suffered from poor DFS when compared with patients with normal GNRI.

### 3.5. Meta-Analysis for PFS

**Figure 5:**
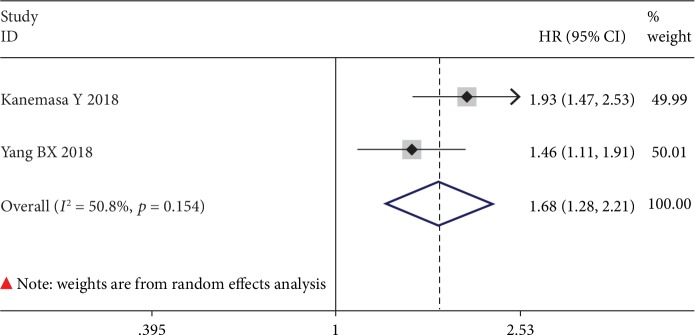
The forest plot for the effect of GNRI on PFS in human malignancies.

The association between GNRI and PFS was investigated by only 2 studies with 725 cases. Heterogeneity could be identified among these 2 studies (*I*^2^ = 50.8%, *p* = 0.154); thus, a random effects model was adopted to perform this meta-analysis. Meta-analysis results evinced that low GNRI was associated with PFS (HR = 1.68, 95% CI: 1.28-2.21, *p* ≤ 0.001) (Figure 5), suggesting that patients with low GNRI suffered from poor PFS when compared with patients with normal GNRI.

### 3.6. Stratification Analysis for the Meta-Analysis with OS

**Figure 6:**
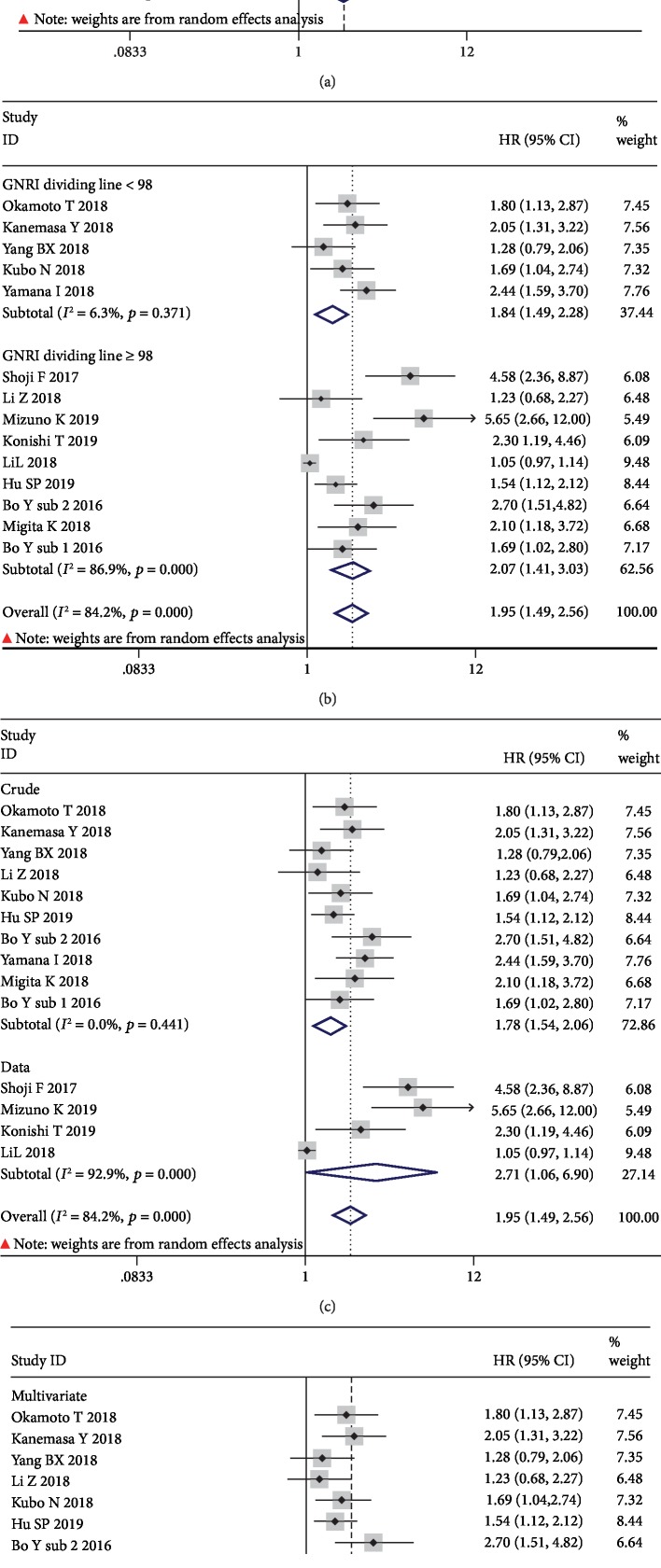
Stratified analysis for the meta-analysis with OS by study sample size (a), GNRI dividing line (b), HR source (c), and analytic method (d).

Considering that more than ten studies were included in the meta-analysis with OS and heterogeneity was identified in this meta-analysis result, thus, stratification analysis was conducted in this process. As shown in Table 2, despite the variation of publishing year, population country, sample capacity, cancer system, primary therapy, GNRI dividing line, HR source, or analytic methodology, the low GNRI was associated with poor OS in human malignancies. Nevertheless, no heterogeneity could be found the subgroup meta-analysis when defined by study sample size < 240 (Figure 6(a)), GNRI value < 98 (Figure 6(b)), HR source from crude origin (Figure 6(c)), or multivariate analysis (Figure 6(d)).

### 3.7. Sensitivity Analyses

**Figure 7:**
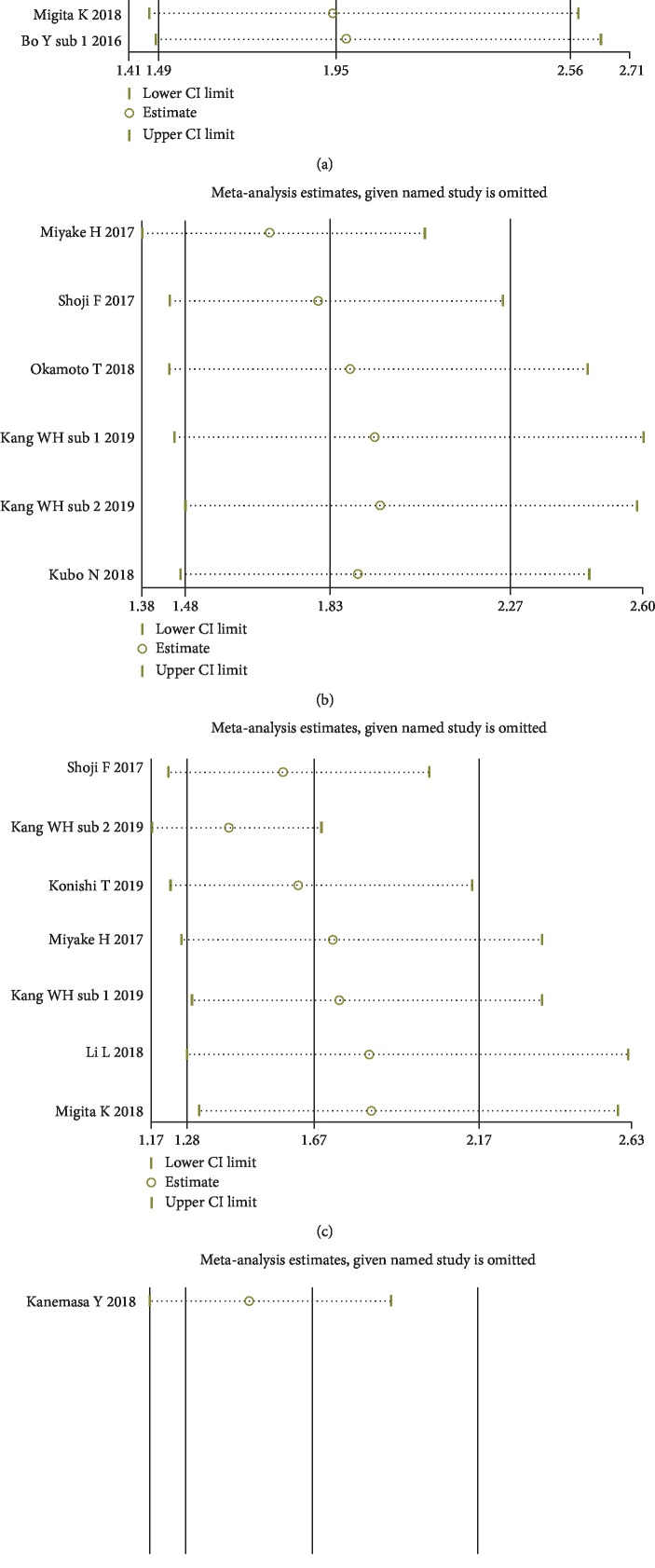
Sensitivity analysis for the correlation of GNRI with OS (a), CSS (b), DFS (c), and PFS (d).

To examine the robustness of our results, sensitivity analysis was performed by removing each individual included study. Omitting any of the included studies did not change the combined meta-analysis effect of GNRI on the HRs for OS, CSS, DFS, or PFS (Figures 7(a)–7(d)). That is to say, our findings were robust across sensitivity analyses.

### 3.8. Publication Bias

For the meta-analysis with OS, no publication bias was found by Begg's test (*p* = 0.155), but publication bias was found by Egger's test (*p* ≤ 0.05). Publication bias was not examined in the other meta-analysis, since the included study number was less than ten.

## 4. Discussion

With the ratio of older people continuing to rise, designing age-specific nutritional policies is a matter of necessity [30]. As a dichotomous index, the GNRI combines two nutritional indicators: albumin and actual weight compared with desirable weight, and the GNRI seems to account for both acute and chronic reasons of nutrition-related outcomes [30]. When compared with Mini Nutritional Assessment (MNA) validated for grading nutritional status in the elderly, the GNRI has been reported to show poor agreement in nutritional assessment but appeared to better predict outcome [31].

To our knowledge, this present meta-analysis firstly evinced that lower GNRI is associated with poor prognosis in human malignancies. Based on current evidence, our meta-analysis exploited 15 studies with 8,046 malignancy cases. Meta-analysis results proved that low GNRI was associated with poor OS, poor CSS, poor DFS, and poor PFS, indicating that malignancy patients with low GNRI would suffer from reduced survival rate and increased recurrence rate. Meanwhile, stratified meta-analysis showed that low GNRI was associated with poor OS, though the publishing year, population country, sample capacity, cancer system, primary therapy, GNRI dividing line, HR source, and analytic methodology varied between different groups.

As a new nutrition-related risk assessment toll, the GNRI was also reported to be associated with postoperative complications in cancer patients (consisting of esophageal cancer, gastric cancer, liver cancer, gallbladder cancer, pancreatic cancer, and colon cancer) after abdominal surgery [2, 32]. Additionally, the preoperative GNRI was also reported to be a risk factor for surgical site infection in patients with soft-tissue sarcoma resection [33]. Taking all these results together, as an indicator of nutritional assessment, the GNRI is associated with adverse outcomes in human malignancies.

The obvious limitation for our meta-analysis is heterogeneity, especially in the meta-analysis with OS and DFS. However, no heterogeneity could be found in the subgroup meta-analysis using the studies defined by study sample size < 240, GNRI value < 98, HR source from crude origin, or multivariate analysis. Due to the small number of included studies in the meta-analysis with DFS, stratified analysis was not conducted in this part. The minor limitation for our meta-analysis is publication bias, which was found in the meta-analysis with OS. Nevertheless, the combined meta-analysis effect of GNRI on the HRs for OS, CSS, DFS, and PFS was not altered during the sensitivity analysis. Finally, we must declare that the data extraction, statistics analysis, and stratified analysis methods used in this research were nearly the same as those used in our team's previously published meta-analysis researches [34, 35]. Therefore, there was a textual overlap between this present research and our previously published researches.

## 5. Conclusion

In summary, GNRI is associated with poor prognosis in human malignancies, and GNRI should be used as a predictive indicator of adverse outcomes during malignancy treatment.

## Figures and Tables

**Table 1:** Summary characteristics of included studies in this meta-analysis.

Author information	Year	Country	Cancer species	No. of patients	Age	Gender ratio	Primary therapy	Cut-off for GNRI (no-risk/risk)	Endpoint	HR and 95% CI	Source of HR	Analytic method	Follow-up interval (months)
Konishi et al. [15]	2019	Japan	Acute myeloid leukemia	63	>50	21/42	Stem cell transplantation	98 (31/32)	OS, DFS	OS HR: 2.30 (1.19-4.46)	Data	Univariate	Median 33.9 (0.6-68.1)
										DFS HR: 2.18 (1.11-4.26)	Data	Univariate	
Mizuno et al. [16]	2019	Japan	Follicular lymphoma	130	Median 67 (32-91)	70/60	Chemotherapy	99.2 (96/34)	OS	OS HR: 5.65 (2.66-12.00)	Data	Univariate	Median 52
Li et al. [17]	2018	China	DLBCL	267	NR	121/156	Chemotherapy	98 (162/105)	OS	OS HR: 0.81 (0.44-1.48)	Crude	Multivariate	Up to 72
Kanemasa et al. [18]	2018	Japan	DLBCL	476	Median 68 (27-97)	210/266	Chemotherapy	96.8 (221/255)	OS, PFS	OS HR: 2.05 (1.31-3.22)	Crude	Multivariate	Median 45
										PFS HR: 1.93 (1.47-2.53)	Data	Univariate	
Yang et al. [19]	2018	China	DLBCL	249	Median 60 (18-85)	107/142	Chemotherapy	96.6 (136/113)	OS, PFS	OS HR: 1.28 (0.79-2.06)	Crude	Multivariate	Median 16 (0-51)
										PFS HR: 1.46 (1.11-1.91)	Data	Univariate	
Yamana et al. [20]	2018	Japan	ESCC	216	NR	NR	Resection	92 (153/63)	OS	OS HR: 2.44 (1.59-3.70)	Crude	Univariate	Up to 60
Kubo N [21]	2018	Japan	EC	240	Mean 63.3 (66-113)	44/196	Resection	92(196/44)	OS, CSS	OS HR: 1.69 (1.04-2.74)	Crude	Multivariate	Up to 60
										CSS HR: 1.60 (0.91-2.82)	Crude	Multivariate	
Migita et al. [22]	2018	Japan	ESCC	137	NR	21/116	Resection	98 (92/45)	OS, DFS	OS HR: 2.10 (1.18-3.72)	Crude	Multivariate	Up to 60
										DFS HR: 1.23 (1.00-1.51)	Data	Univariate	
Bo et al. [23]	2016	China	ESCC	239	Mean 67.9 (60-88)	89/150	Radiotherapy	98 (184/55)	OS	OS HR: 1.69 (1.02-2.80) sub1	Crude	Multivariate	Up to 60
										OS HR: 2.70 (1.51-4.82) sub2	Crude	Multivariate	
Li et al. [24]	2018	China	Hepatocellular carcinoma	261	Median 68 (67-70)	46/215	Resection	98 (197/64)	OS, DFS	OS HR: 1.05 (0.97-1.14)	Data	Univariate	Up to 60
										DFS HR: 1.29 (1.06-1.57)	Data	Univariate	
Hu et al. [25]	2019	China	Pancreatic cancer	265	NR	109/156	Resection	98 (170/95)	OS	OS HR: 1.54 (1.12-2.12)	Crude	Multivariate	Up to 60
Miyake et al. [26]	2017	Japan	Renal cell carcinoma	432	NR	155/277	Resection	98 (325/107)	CSS, DFS	CSS HR: 3.37 (1.86-6.14)	Data	Univariate	Up to 100
										DFS HR: 1.52 (1.04-2.22)	Data	Univariate	
Kang et al. [27]	2019	Korea	Renal cell carcinoma	4591	NR	1224/3367	Resection	98 (3859/732)	CSS, DFS	CSS HR: 1.67 (1.19-2.34) sub1	Crude	Multivariate	Median 37 (18-68)
										CSS HR: 1.61 (1.13-2.28) sub2	Crude	Multivariate	
										DFS HR: 1.33 (0.82-2.17) sub1	Crude	Multivariate	
										DFS HR: 3.18 (2.00-5.06) sub2	Crude	Multivariate	
Okamoto et al. [28]	2018	Japan	Prostate cancer	339	Median 72	0/339	Androgen-deprivation	92 (273/66)	OS, CSS	OS HR: 1.80 (1.13-2.87)	Crude	Multivariate	Median 26 (12-53)
										CSS HR: 1.76 (1.04-2.98)	Crude	Multivariate	
Shoji et al. [29]	2017	Japan	Lung cancer	141	Median 68 (37-86)	80/61	Resection	98 (119/22)	OS, CSS, DFS	OS HR: 4.58 (2.36-8.87)	Data	Univariate	Median 58 (0-94)
										CSS HR: 3.09 (0.98-9.68)	Data	Univariate	
										DFS HR: 4.03 (1.45-10.73)	Crude	Multivariate	

Abbreviations: DLBCL: diffuse large B cell lymphoma; NR: no reported; ESCC: esophageal squamous cell carcinoma; EC: esophageal cancer; OS: overall survival; CSS: cancer-specific survival; DFS: disease-free survival; PFS: progression-free survival; HR: hazard ratio.

**Table 2:** Stratification analysis for the meta-analysis with overall survival (OS) in patients with malignancies.

Subgroup	No. of studies	No. of patients without risk	No. of patients at risk	Pooled HR (95% CI)	Heterogeneity	
					*I* ^2^ (%)	*p* value
Altogether	13	2,030	993	2.01 (1.49-2.71)	85.0	≤0.001
Publishing time						
≥2018	10	1,565	811	1.86 (1.38-2.51)	84.4	≤0.001
<2018	3	465	182	2.21 (1.31-3.75)	69.3	0.021
Country						
Japan	8	1,181	561	2.40 (1.86-3.10)	44.7	0.081
China	5	849	432	1.43 (1.08-1.90)	72.2	0.031
Sample capacity						
≥240	7	1,355	742	1.45 (1.13-1.85)	70.5	0.002
<240	6	675	251	2.66 (2.00-3.55)	42.6	0.107
Dividing line for GNRI						
≥98	8	1,051	452	2.07 (1.41-3.03)	86.9	≤0.001
<98	5	979	541	1.84 (1.49-2.28)	6.3	0.371
Cancer system						
Hematological	5	646	539	2.02 (1.27-3.20)	69.4	0.011
Digestive	6	992	366	1.74 (1.25-2.43)	84.0	≤0.001
Urinary	1	273	66	1.80 (1.13-2.87)	—	—
Respiratory	1	119	22	4.58 (2.36-8.88)	—	—
Primary therapy						
Resection	6	927	333	1.90 (1.25-2.88)	88.6	≤0.001
Chemotherapy	4	615	507	1.97 (1.12-3.49)	76.3	0.005
Radiotherapy	1	184	55	2.09 (1.32-3.30)	—	—
Others	2	304	98	1.95 (1.33-2.86)	0.0	0.552
HR source						
Crude	9	1,587	841	1.78 (1.54-2.06)	0.00	0.441
Data	4	443	152	2.71 (1.06-6.90)	92.9	≤0.001
Analytic method						
Univariate	5	596	215	2.62 (1.28-5.35)	92.7	≤0.001
Multivariate	8	1,434	778	1.70 (1.46-1.99)	0.0	0.590

## Data Availability

The data supporting this meta-analysis are from previously reported studies, which have been cited. The processed data are available from the corresponding author upon request.
